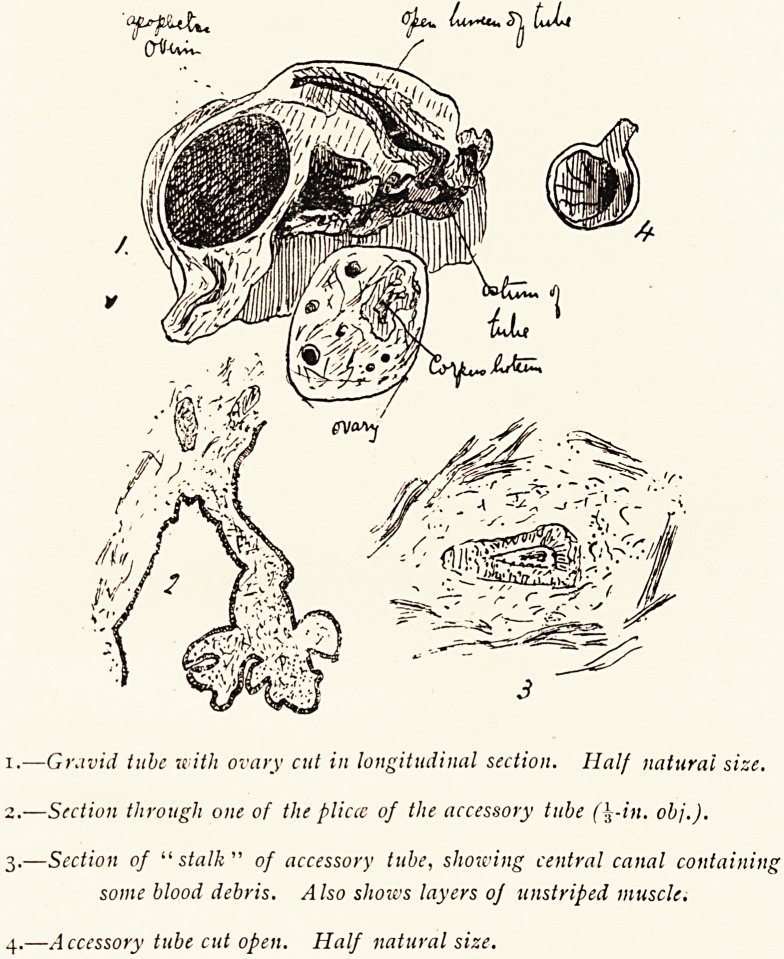# A Case of Tubal Gestation Producing Severe Hemorrhage without Rupture, Associated with the Presence of an Accessory Fallopian Tube

**Published:** 1904-03

**Authors:** Ernest W. Hey Groves

**Affiliations:** Assistant-Surgeon to the Bristol General Hospital


### A CASE OF TUBAL GESTATION PRODUCING SEVERE HEMORRHAGE WITHOUT RUPTURE, ASSOCIATED WITH THE PRESENCE OF AN ACCESSORY FALLOPIAN TUBE.

BY

ERNEST W. HEY GROVES, M.D., B.Sc. Lond.,

*Assistant-Surgeon to the Bristol General Hospital.*

---

THE following case of extra-uterine gestation presents several points of great interest, both clinically and pathologically, which make it worth recording.

The patient, a married woman, aged 24, had had one child three years before. Her last regular period was January 28th, 1903: it lasted four days. Nothing further until March 9th, when an irregular uterine bleeding began and continued until the operation, March 25th.

March 20th.—Began to have intermittent pains, chiefly in the right lower abdomen, which got worse for the next three days. The breasts have become full and tender.

March 23rd.—Pain has become much more severe, and patient feels very ill. A swelling and resistance can be seen and felt extending three fingers' breadth above pubes. Cervix is pushed forwards. Vagina and cervix are dark blue. There is a soft irregular fulness in Douglas's pouch, extending up behind and to the right of the uterus. Uterine sound passed 2½ inches with the concavity forward in front of the swelling. The pulse was 124; temp. 101° F.

She was kept in bed and watched for thirty-six hours, but the swelling and pain becoming worse, and the pulse and temperature remaining about 120 and 101° F., it was decided to operate, as it seemed clearly a case of extra-uterine gestation in which hemorrhage was continuing in spite of rest.

March 25th.—Laparotomy. A large quantity of dark semi-liquid blood-clot, together with some quite fluid blood, was found in the lower abdomen and filling up the pelvis, which was roofed over by an unusually large sigmoid flexure of the colon. The right Fallopian tube was enlarged and soft, and its distal end was buried in the deep portions of blood-clot behind the uterus. The right tube, ovary, broad ligament and all loose clot were removed. The pelvis was swabbed out with dry sponges and the wound closed without drainage. The patient recovered without any drawback, and is now quite well.

The specimen, which has been cut open after hardening by Kaiserling's method, shows the ampulla of the right Fallopian tube distended by a mole in which disorganised embryonic structures can be distinctly seen. The outer third of the tube is swollen, but its lumen is quite distinct, and opens by a wide and patent ostium surrounded by engorged fimbriæ. No rupture of the tube is found either towards the peritoneum or the broad ligament. The ovary shows a well-marked corpus luteum of pregnancy.

It is evident that the hemorrhage could only have occurred by a gradual "blood-drip" from the open ostium of the tube. It is now well recognised that the hemorrhage in the early stages of tubal gestation is brought about, not by mere mechanical distention and rupture of the tube and of blood vessels, but by an erosion of the tubal walls and vessels by the embryonic trophoblast. The ovum, in fact, does not occupy the lumen of the tube, but embeds itself in the wall of the tube in the sub-mucous layer. And it is the scantiness of this layer and its inability to provide the adequate amount of decidua that leads either to the opening up of blood vessels and the formation of a mole or the perforation into the peritoneal cavity. Abundant evidence and illustrations of these facts are brought forward by Andrews in the *Journal of Obstetrics and Gynæcology* for 1903.

In addition to the gravid tube two other bodies were found in the blood-clot. One, about the size and shape of a small grape, proved to be an encapsuled blood-clot surrounded by organised fibrin. This was probably the first blood which escaped from the tube, and shows that hemorrhage must have begun in small quantities some time before symptoms developed.

The other, which is figured of proportionate size, is a stalked globular mass about three-quarters of an inch in diameter. It is lined by a smooth membrane which is thrown into several distinct folds. The wall of this little body consists of unstriped muscle and loose areolar tissue. The lining membrane consists of one or two layers of columnar epithelium which is produced into miniature plicæ exactly similar to the plicæ in a Fallopian tube. The apparently solid stalk has a central cavity which contains some free blood corpuscles. This cavity is also lined by elongated columnar cells. The structure is an accessory Fallopian tube, which Kussmann has shown to be the remainder of the pronephric tubules. The presence of free blood in the lumen of the stalk of this accessory tube probably shows that it was connected with the lumen of the gravid tube when the latter was filled with blood, and it was detached in the manipulations of the operation.

Three somewhat similar cases of dilated accessory Fallopian tubes are figured and carefully described from the Museum of the Royal College of Surgeons by Sampson Handley in the *Journal of Obstetrics and Gynæcology* for 1903.

In conclusion, it is of considerable interest to note the association of tubal gestation with the occurrence of a congenital malformation, such as the presence of an accessory Fallopian tube.

1.—*Gravid tube with ovary cut in longitudinal section. Half natural size.*

2.—*Section through one of the plicæ of the accessory tube ($\frac{1}{3}$-in. obj.).*

3.—*Section of "stalk" of accessory tube, showing central canal containing some blood debris. Also shows layers of unstriped muscle.*

4.—*Accessory tube cut open. Half natural size.*

## Figures and Tables

**1. 2. 3. 4. f1:**